# NFKB2 polymorphisms associate with the risk of developing rheumatoid arthritis and response to TNF inhibitors: Results from the REPAIR consortium

**DOI:** 10.1038/s41598-020-61331-5

**Published:** 2020-03-09

**Authors:** Jose Manuel Sánchez-Maldonado, Manuel Martínez-Bueno, Helena Canhão, Rob ter Horst, Sonia Muñoz-Peña, Ana Moñiz-Díez, Ana Rodríguez-Ramos, Alejandro Escudero, Signe B. Sorensen, Merete L. Hetland, Miguel A. Ferrer, Bente Glintborg, Ileana Filipescu, Eva Pérez-Pampin, Pablo Conesa-Zamora, Antonio García, Alfons den Broeder, Salvatore De Vita, Svend Erik Hove Jacobsen, Eduardo Collantes, Luca Quartuccio, Mihai G. Netea, Yang Li, João E. Fonseca, Manuel Jurado, Miguel Ángel López-Nevot, Marieke J. H. Coenen, Vibeke Andersen, Rafael Cáliz, Juan Sainz

**Affiliations:** 1 Genomic Oncology Area, GENYO. Centre for Genomics and Oncological Research: Pfizer/University of Granada/Andalusian Regional Government, PTS Granada, Granada, Spain; 2 Instituto de Investigación Biosanataria IBs.Granada, Granada, Spain; 3 Area of Genomic Medicine, GENYO. Centre for Genomics and Oncological Research: Pfizer/University of Granada/Andalusian Regional Government, Granada, Spain; 4 CEDOC, EpiDoC Unit, NOVA Medical School and National School of Public Health, Universidade Nova de Lisboa, Lisbon, Portugal; 5 Department of Internal Medicine and Radboud Center for Infectious Diseases, Radboud University Nijmegen Medical Center, Nijmegen, The Netherlands; 6 Department for Immunology & Metabolism, Life and Medical Sciences Institute (LIMES), University of Bonn, 53115 Bonn, Germany; 7 Department of Genetics, University of Groningen, University Medical Center Groningen, Groningen, The Netherlands; 8 Rheumatology department, Reina Sofía Hospital/IMIBIC/University of Córdoba, Córdoba, Spain; 9 Focused research unit for Molecular Diagnostic and Clinical Research, IRS-Center Sonderjylland, Hospital of Southern Jutland, DK-6200 Aabenraa, Denmark; 10 Institute of Molecular Medicine, Faculty of Health Sciences, University of Southern Denmark, Odense, Denmark; 11 The DANBIO registry, The Danish Rheumatologic Biobank and Copenhagen Center for Arthritis Research (COPECARE), Center for Rheumatology ad Spine Diseases, Centre of Head and Orthopaedics, Rigshospitalet, Glostrup, Denmark; 12 Department of Clinical, Faculty of Health and Medical Sciences, University of Copenhagen, Copenhagen, Denmark; 13 Rheumatology department, Virgen de las Nieves University Hospital, Granada, Spain; 14 Rheumatology department, University of Medicine and Pharmacy “Iuliu Hatieganu”, Cluj-Napoca, Romania; 15 Rheumatology Unit, University Hospital of Santiago de Compostela, Santiago de Compostela, Spain; 16 Clinical Analysis department, Santa Lucía University Hospital, Cartagena, Spain; 17 Radboud university medical center, Radboud Institute for Health Sciences, Department of Human Genetics, Nijmegen, The Netherlands; 18 Department of Medical Area, Clinic of Rheumatology, University of Udine, Udine, Italy; 19 Department of Biochemistry and Immunology. University Hospital of Southern Jutland, Jutland, Denmark; 20 Rheumatology and Metabolic Bone Diseases Department, Hospital de Santa Maria, CHLN, Lisbon, Portugal; 21 Rheumatology Research Unit, Instituto de Medicina Molecular, Faculty of Medicine, University of Lisbon, Lisbon Academic Medical Center, Lisbon, Portugal; 22 Immunology department. Virgen de las Nieves University Hospital, Granada, Spain

**Keywords:** Diagnostic markers, Prognostic markers, Rheumatoid arthritis

## Abstract

This study sought to evaluate the association of 28 single nucleotide polymorphisms (SNPs) within NFKB and inflammasome pathway genes with the risk of rheumatoid arthritis (RA) and response to TNF inhibitors (TNFi). We conducted a case-control study in a European population of 1194 RA patients and 1328 healthy controls. The association of potentially interesting markers was validated with data from the DANBIO (695 RA patients and 978 healthy controls) and DREAM (882 RA patients) registries. The meta-analysis of our data with those from the DANBIO registry confirmed that anti-citrullinated protein antibodies (ACPA)-positive subjects carrying the NFKB2_rs11574851T_ allele had a significantly increased risk of developing RA (PMeta_ACPA + = 0.0006) whereas no significant effect was found in ACPA-negative individuals (PMeta_ACPA− = 0.35). An ACPA-stratified haplotype analysis including both cohorts (n = 4210) confirmed that ACPA-positive subjects carrying the NFKB2_TT_ haplotype had an increased risk of RA (OR = 1.39, P = 0.0042) whereas no effect was found in ACPA-negative subjects (OR = 1.04, P = 0.82). The meta-analysis of our data with those from the DANBIO and DREAM registries also revealed a suggestive association of the NFKB2_rs1056890_ SNP with larger changes in DAS28 (OR = 1.18, P = 0.007). Functional experiments showed that peripheral blood mononuclear cells from carriers of the NFKB2_rs1005044C_ allele (in LD with the rs1056890, r2 = 1.00) showed increased production of IL10 after stimulation with LPS (P = 0.0026). These results provide first evidence of a role of the NFKB2 locus in modulating the risk of RA in an ACPA-dependent manner and suggest its implication in determining the response to TNFi. Additional studies are now warranted to further validate these findings.

## Introduction

Rheumatoid arthritis (RA) is a chronic inflammatory disease more frequently diagnosed in females than males, that has a prevalence of about 0.5–1%^1^. RA perpetuates and amplifies itself through a wide number of molecular mechanisms involving several immune cell types and multiple inflammatory mediators that are released from the damaged tissue^2^. Although the complexity of inflammatory pathways implicated in RA development and progression remains in part unknown, there are convincing evidences supporting the view that NFKB pathway and its connection with the NLRP3-inflammasome plays a pivotal role in the modulation of the expression of multiple inflammatory genes implicated in RA development^3^ and drug response or disease progression^4^.

Activated NFKB has been detected in the synovium of RA patients at both early and late stages of joint inflammation^5–8^ and once NFKB is activated (for instance, through the interaction of antigen presenting cells and T cells), it triggers two major signaling pathways in the implicated cells: the canonical and the non-canonical NFKB pathway. Whereas the canonical pathway regulates the activation of *NFKB1 p50*, *RELA* and *c-REL* and leads to rapid but transient NFKB activation, the non-canonical NFKB pathway selectively activates p100-sequestered NFKB members (predominantly *NFKB*2 *p5*2 and *RELB*) and produces a long-lasting signaling. Even though a cross-talk between the canonical and non-canonical NFKB pathways has been previously reported, the activation of the canonical NFKB pathway is generally associated with inflammation whereas the induction of the non-canonical NFKB pathway was linked to development processes^9^. In RA, it is well known that the acute activation of the canonical pathway on antigen presenting cells and T cells quickly leads to the production of a wide range of essential proinflammatory mediators including cytokines (TNFα, IL1α, IL1β, IL1RA, IL2, IL12p40 and IFNγ), chemokines (IL8, CXCL11), immunoreceptors (CD80, CD23, CD48, CD69, IL2R, TNFRs, and CCR5), cell adhesion molecules (ELAM-1, ICAM-1, VCAM-1 and P-selectin) and growth factors (GM-CSF, IGFBP2, and PDGFB) that often facilitate synovial hyperplasia by promoting cell proliferation and apoptosis inhibition of RA fibroblast-like synovial cells^10^. On the contrary, the activation of the non-canonical pathway involves a slow build-up of long-lasting signals that have been implicated in developmental processes including B-cell development^11^, secondary lymphoid organ development^12,13^ and osteoclast differentiation^14^ but also development of myeloid-related CD4^+^CD8α^−^ dendritic cells and macrophages^15^, key players in modulating immune responses in RA.

Besides the role of NFKB in the inflammatory process, recent evidences have shown that the NLRP3-inflammasome is a cytosolic multiprotein complex highly expressed in peripheral blood mononuclear cells of RA patients and in the synovial tissues of osteoarthritis patients. The NLRP3 inflammasome is capable of alerting immune system to the presence of tissue damage and to induce the processing of the IL1β, IL18 and IL33 pro-cytokines into biologically active proinflammatory mediators that drive cartilage destruction^16^. In addition, it has been reported that the presence of mutations in NLRP3-inflammasome-related proteins (CARD8 and NLRP3) predispose to RA^17,18^ and that genetic variation in this pathway might also modulate inflammatory activity in early stages of the disease and thereby affect disease progression^17,18^.

Considering the aspects detailed above, but also previous studies suggesting that the NFKB- and NLRP3-inflammasome pathways are genetically determined^19^, we decided to conduct a case-control study to investigate whether 28 single nucleotide polymorphisms (SNPs) within the *NFKB1*, *NFKB2*, *NFKBIB*, *IKBKB*, *GBP6*, *IRF4*, *NLRP3*, *REL*, *RELA*, *KLRC1*, *KLRK1* | *KLRC4*, *LOC105376246* (ncRNA), *TLR4*, *TLR5*, *TLR9*, *TLR10* and *TRAF1* | *C5* genes influence the risk of developing RA and the response to TNF inhibitors (TNFi). In addition, we investigated the correlation of selected SNPs with steroid hormone levels and their role in modulating immune responses after stimulation of whole blood, peripheral mononuclear cells (PBMCs) and macrophages with lipopolysaccharide (LPS), phytohemagglutinin (PHA) and Pam3Cys.

## Material and Methods

### Discovery population

The discovery population consisted of 1194 RA patients and 1328 healthy controls ascertained through the REPAIR consortium (Table 1). RA patients fulfilled the 1987 revised American College of Rheumatology (ACR)^20^ and the ACR/EULAR 2010 classification criteria^21^. The study followed the Declaration of Helsinki. Study participants were of European origin and gave their written informed consent to participate in the study, which was approved by the ethical review committee of participant institutions. The Ethics committee of each participant institution approved the study protocol: Virgen de las Nieves University Hospital (2012/89); Santa Maria Hospital-CHLN (CE 877/121.2012); University Clinical Hospital of Santiago de Compostela (2013/156). A detailed description of the discovery population has been reported elsewhere^22–24^.

**Table 1:** Demographic and clinical characteristics of RA patients.

RA patient populations			
Demographic characteristics	Discovery Population (n = 1194)	DREAM ^†^ Registry (n = 882)	DANBIO Registry (n = 695)
*Age (years)*	59.22 ± 12.97	54.63 ± 12.80	54.27 ± 13.30
*Gender ratio (female/male)*	4.01 (959/234)	2.07 (477/230)	2.80 (512/183)
**Clinical assessment**			
RF positive patients*	764 (68.64)	534 (77.62)	221 (64.06)
ACPA positive patients*	643 (70.74)	151 (62.14)	390 (72.90)
*DAS28 at baseline*	5.74 ± 2.15	5.33 ± 1.26	4.77 ± 1.23
*Disease duration (years)*	17.60 ± 9.99	9.70 ± 9.57	7.89 ± 8.86
**Treatments**			
*csDMARDs at baseline*			
*Methotrexate (%)*	798 (66.83)	463 (65.40)	514 (73.95)
*Leflunomide (%)*	324 (27.14)	ND	ND
*Sulphasalazine (%)*	149 (12.48)	ND	ND
*First biologic agent*			
*Infliximab (%)*	386 (32.33)	244 (34.46)	159 (22.88)
*Etanercept (%)*	227 (19.01)	130 (18.36)	200 (28.78)
*Adalimumab (%)*	191 (16.00)	334 (47.18)	173 (24.89)
*Golimumab (%)*	17 (01.42)	—	47 (06.76)
*Certolizumab (%)*	—	—	72 (10.36)
*Rituximab (%)*	13 (01.09)	—	16 (02.30)
*Tocilizumab (%)*	6 (00.50)	—	19 (02.73)
*Anakinra (%)*	—	—	2 (00.29)
*Others (%)*	14 (01.17)	—	7 (01.01)

Data are means ± standard deviation or n (%). Abbreviations: RF, rheumatoid factor; ACPA: anti-cyclic citrullinated peptide antibodies; DAS28, disease activity score; csDMARDs, conventional synthetic disease-modifying antirheumatic drugs. ND, not determined (unknown).

^†^Clinical data for 708 RA patients that were available for genotyping.

*RF was available for 1113, 688 and 345 patients in the discovery, DREAM and DANBIO populations, respectively.

*ACPA was available for 908, 127 and 535 patients in the discovery, DREAM and DANBIO populations, respectively.

### Response to anti-TNF medications

Six hundred and four RA patients treated with TNFi (adalimumab, etanercept, infliximab, golimumab or certolizumab) were included in the drug response analysis of the discovery population. The change in disease activity score (DAS28) at baseline and at 6 months of treatment with TNFi was calculated for each patient. Linear regression analysis adjusted for age and sex was used to determine the association between selected SNPs and changes in DAS28. Subjects with missing values for DAS28 in any of these time points were not included in the analysis.

### SNP selection and genotyping

NFKB- and inflammasome-related polymorphisms were selected on the basis of their potential functionality and linkage disequilibrium (LD) but also because of existing studies reporting their significant association with the risk of developing autoimmune and immune-related diseases or response to TNFi^25–29^. This strategy resulted in the selection of 28 genetic variants within the *GBP6*, *IKBKB*, *IRF4*, *KLRC1*, *KLRK1*, *NFKB1*, *NFKB2*, *NFKBIB*, *NLRP3*, *REL*, *RELA*, *RELB*, *TLR4*, *TLR5*, *TLR9*, *TLR10* and *TRAF1/C5* loci that were genotyped in the discovery population (Table 2). Genomic DNA was extracted from peripheral blood using the Qiagen Mini Kit (Qiagen, CA, USA) or from saliva using standard procedures. Genotyping was carried out using KASPar® assays (LGC Genomics, London, UK) in a 384-well plate format (Applied Biosystems, CA, USA) according to manufacturer’s instructions. Five percent of samples were included as duplicates to ensure high-quality genotyping.

**Table 2:** Selected SNPs within NFKB-related genes.

Gene	Chr.	dbSNP rs#	Nucleotide substitution	Effect-allele	Location	Reported associations with autoimmune diseases, drug response and/or potential functional role
*GBP6*	1	rs928655	A/G	A	Intronic	Associated with etanercept response in moderate-to-severe plaque psoriasis^47^
*IKBKB*	8	rs11986055	A/C	A	Intronic	
*IRF4*	6	rs1050975	A/G	A	3′-UTR/ncRNA	
*IRF4*	6	rs12203592	C/T	T	Intronic	Correlated with white blood cell count^48^
*IRF4*	6	rs1877175	C/T	T	3′-UTR/ncRNA	
*IRF4*	6	rs7768807	T/C	T	3′-UTR/ncRNA	
*KLRC1*	12	rs7301582	C/T	T	Intronic	Associated with response to anti-TNF therapy in RA patients^49^
*KLRK1* | *KLRC4*	12	rs1049174	C/G	C	3′UTR/Intronic	Associated with response to anti-TNF therapy in RA patients^50^
*KLRK1* | *KLRC4*	12	rs1154831	A/C	A	Intronic/Near gene	Lack of association with response to anti-TNF therapy^50^
*KLRK1* | *KLRC4*	12	rs2255336	A/G	A	Thre72Ala	Correlation with blood NKG2D type II integral membrane protein levels^51^ and associated with response to anti-TNF therapy in RA patients^50^; Associated with a decreased risk of Lupus erythematosus^52,53^
*LOC105376246*	9	rs2722824	A/C	A	Near gene	
*NFKB1*	4	rs4648110	A/T	A	Intronic	
*NFKB2*	10	rs11574851	C/T	T	Asn698Asn	
*NFKB2*	10	rs12769316	C/T	T	Near gene	
*NFKB2* | *PSD*	10	rs1056890	C/T	T	Near gene/3′-UTR	
*NFKBIB*	19	rs3136645	C/T	C	ncRNA	Associated with response to anti-TNF drugs in RA patients^25^
*NLRP3*	1	rs4612666	C/T	T	Intronic	Associated with response to anti-TNF drugs in RA patients^42^
*REL*	2	rs13031237	G/T	T	Intronic	Overall association with the risk of RA at GWAS level^29,54,55^. Association with RA in ACPA-positive individuals at GWAS level^55^; Association with early-onset psoriasis^56^ and autoimmune diseases^57^ in large candidate gene association studies
*REL*	2	rs842647	A/G	A	Intronic	Associated with susceptibility to Behcet’s disease^58^
*REL*	2	rs13017599	A/G	A	Near gene	Associated with RA and psoriatic arthritis at GWAS level^29,59^ and in a candidate gene association study^60^
*RELA*	11	rs11820062	C/T	T	Intronic	Eosinophil counts^48^
*RELA*	11	rs2306365	A/G	A	Intronic	
*RELA*	11	rs7119750	C/T	T	Intronic	
*TLR10*	4	rs11096957	A/C	A	Asn241His	Associated with hip osteoarthritis^61,62^ and effectiveness of biologics for psoriasis treatment at GWAS level^63^
*TLR4*	9	rs4986791	C/T	T	Thr399Ile	TLR4: lymphocyte 96 antigen complex level^51^; Associated with RA risk and response to anti-TNF drugs^64^; Associated with risk of developing inflammatory bowel disease^65^
*TLR5*	1	rs5744174	C/T	C	Phe616Leu	Associated with response to anti-TNF drugs in RA patients^27^; Associated with the risk of Crohns disease^66^ and response to anti-TNF treatment^67^; Associated with response to ustekinumab treatment in psoriasis patients^68^
*TLR9* | | *TWF2*	3	rs187084	G/A	T	Near gene	Associated with psoriatic arthritis risk^69^, hip and knee osteoarthritis^70,71^, SLE^72^ and IBD^73^; associated with the risk of autoimmune thyroid disease^74^; response to anti-TNF therapy in patients with RA^64^ and IBD^75^
*TRAF1* | | *C5*	9	rs3761847	A/G	A	Near gene	Associated with RA at GWAS level^54,76^

Abbreviations: SNP, single nucleotide polymorphism; UTR, untranslated region; ncRNA, non-coding Ribonucleic acid. Risk alleles were select according to available GWAS data in order to make possible a meta-analysis of the discovery and replication cohorts.

### Statistical analysis

The Hardy-Weinberg Equilibrium (HWE) test was performed in the control group by a standard observed-expected chi-square (χ^2^). Logistic and linear regression analyses adjusted for age, sex and country of origin were used to assess the main effects of the selected SNPs on RA risk and the response to TNFi respectively. Statistical power was estimated using Quanto software (http://hydra.usc.edu/gxe/). Correction for multiple testing was performed using the Meff method for SNPs genotyped across all populations^30^. The threshold used for the risk and drug response analyses was 0.0008 ([0.05/22 independent markersx3 inheritance models).

### Linkage disequilibrium (LD) and haplotype analysis

We performed haplotype frequency estimation and haplotype association analysis adjusted for age, sex and country of origin using SNPstats^31^ and haplo.stats package in STATA. Haplotype frequencies were determined using the Expectation-maximization (EM) algorithm. Haplotypes were reconstructed using SNPtool and Haploview and block structures were determined according to the method of Gabriel *et al*.^32^.

### Replication populations and meta-analyses for RA risk and drug response

For replication purposes, we genotyped the most promising SNPs associated with RA risk in a cohort of 695 Danish RA patients and 978 healthy controls^33^. Clinical data from RA patients were collected through the DANBIO registry (The National Danish Registry for Biological Treatment of Rheumatic Diseases)^34^ and DNA samples were obtained from peripheral blood collected at the Statens Serum Institut (Copenhagen, Denmark), which routinely perform screening for tuberculosis before treatment with biological treatments. Healthy blood donors were recruited in Viborg and Sønderborg (Denmark). In order to replicate the most interesting associations with response to TNFi, we also used genetic data from a genome-wide association study (GWAS) on drug response conducted in 882 Dutch RA patients from the DREAM (Dutch RhEumatoid Arthritis Monitoring) registry. Imputed SNPs reporting potentially interesting overall or ACPA-specific associations with RA risk or drug response were genotyped in a subset of 708 patients. To further validate our results, we also genotyped the most interesting markers associated with drug response in 555 RA patients from the DANBIO registry that were treated with TNFi. A total of 2107 patients were treated with anti-TNF. Demographic and clinical details of the 3 cohorts are included in Supplementary Table 1. The study was approved by the Institutional review board of the Radboud university medical centre and by the Regional Ethics Committee of Central Denmark Region (M-20100153 and S-20120113). All patients provided written informed consent and clinical information was prospectively gathered from the medical records.

To test for genetic association, we conducted a meta-analysis of the discovery data with those from the 2 European registries and the I^2^ statistic was used to assess statistical heterogeneity between studies. The pooled OR was computed using the random-effect model.

### Functional analysis of the NFKB and inflammasome-related variants

Cytokine stimulation experiments were conducted in the 500 Functional Genomics (500FG) cohort from the Human Functional Genomics Project (HFGP; http://www.humanfunctionalgenomics.org/), which was designed to determine the influence of genomic variation on the variability of immune responses. The HFGP study was approved by the Arnhem-Nijmegen Ethical Committee (no. 42561.091.12) and biological specimens (venous blood) were collected after informed consent was obtained. We assessed whether any of the 28 NFKB and inflammasome-related SNPs correlated with cytokine levels (TNFα, IFNγ, IL1β, IL1RA, IL6, IL8, IL10, IL17, and IL22) after the stimulation of whole blood, peripheral blood mononuclear cells (PBMCs) or monocyte-derived macrophages from 408 healthy subjects with LPS (1 or 100 ng/ml), PHA (10 μg/ml), and Pam3Cys (10 μg/ml). After log transformation, linear regression analyses adjusted for age and sex were used to determine the correlation of selected SNPs with cytokine expression quantitative trait loci (cQTLs). All analyses were performed using R software (http://www.r-project.org/). In order to account for multiple comparisons, we used a significant threshold of 0.00025, i.e. 0.05/(22 independent SNPs × 9 cytokines).

Details on PBMCs isolation, macrophage differentiation and stimulation assays have been reported elsewhere^35–37^. Briefly, PBMCs were washed twice in saline and suspended in medium (RPMI 1640) supplemented with gentamicin (10 mg/mL), L-glutamine (10 mM) and pyruvate (10 mM). PBMC stimulations were performed with 5 × 10^5^ cells/well in round-bottom 96-wells plates (Greiner) for 24 hours in the presence of 10% human pool serum at 37 °C and 5% CO_2_. Supernatants were collected and stored in −20 °C until used for ELISA. LPS (100 ng/ml), PHA (10 μg/ml) and Pam3Cys (10 μg/ml) were used as stimulators for 24 or 48 hours. Whole blood stimulation experiments were conducted using 100 μl of heparin blood that was added to a 48 well plate and subsequently stimulated with 400 μl of LPS and PHA (final volume 500 ul) for 48 hours at 37 °C and 5% CO_2_. Supernatants were collected and stored in −20 °C until used for ELISA. Concentrations of human TNFα, IFNγ, IL1β, IL1RA, IL6, IL8, IL10, IL17, and IL22 were determined using specific commercial ELISA kits (PeliKine Compact, Amsterdam, or R&D Systems), in accordance with the manufacturer’s instructions.

Once we examined the correlation of NFKB and inflammasome-related polymorphisms with cytokine levels in our functional experiments, we also used the HaploReg SNP annotation tool (http://www.broadinstitute.org/mammals/haploreg/haploreg.php) to further investigate the functional consequences of each specific variant. We also assessed whether any of the potentially interesting markers correlated with mRNA expression levels of their respective genes using data from GTex portal (www.gtexportal.org/home/).

### Correlation between steroid hormone levels and NFKB- and inflammasome-related SNPs

We also measured serum levels of seven steroid hormones (androstenedione, cortisol, 11-deoxy-cortisol, 17-hydroxy progesterone, progesterone, testosterone and 25 hydroxy vitamin D3) in the 500FG cohort, which includes 531 healthy subjects. Steroid hormones were analyzed by Liquid Chromatography Tandem-Mass Spectrometry (LCMSMS) after protein precipitation and solid-phase extraction as described in Ter Horst *et al*.^37^ (see also Supplementary Material). Hormone levels and genotyping data were available for a total of 406 subjects.

After log-transform, correlation between steroid hormone levels and NFKB- and inflammasome-related SNPs was evaluated by linear regression analysis adjusted for age and sex. In order to avoid a possible bias, we excluded those subjects that were using oral contraceptives or those subjects in which this information was not known from the analysis. A total of 379 healthy subjects (107 women and 272 men) were finally available for analysis. A Bonferroni significance threshold was set to 0.00033 considering the number of independent SNPs tested (n = 22) and the number of hormones determined (n = 7).

## Results

This study was conducted in a discovery population comprised of 1194 RA patients and 1328 healthy controls. RA patients were slightly older than controls (59.22 ± 12.97 vs. 52.67 ± 8.99) and showed an increased female/male ratio compared to healthy controls (4.10 [959/234] vs. 1.39 [773/555]. Sixty percent of the RA patients presented positive values of anti-citrullinated protein antibodies (ACPA) and the median disease duration was of 17.60 years and the disease activity score 28 (DAS28) calculated at patient recruitment was of 5.74 (Table 1).

### Association of selected SNPs with RA risk

All SNPs were in Hardy-Weinberg equilibrium in the control group (P > 0.001). Logistic regression analysis adjusted for age, sex and country of origin showed that carriers of the *NLRP3*_rs4612666T_ allele or the *IRF4*_rs1050975A/A_ and *NFKB2*_rs12769316T/T_ genotypes had an increased risk of developing RA at nominal level of *P* ≤ 0.05 (OR_Dom_ = 1.25, 95%CI 1.05–1.49, P = 0.013; OR_Rec_ = 1.30, 95%CI 1.04–1.62, P = 0.019; and OR_Rec_ = 1.70, 95%CI 1.04–2.78, P = 0.034; Table 3). Interestingly, an ACPA-stratified analysis revealed that ACPA-positive subjects carrying the *NFKB1*_rs4648110A/A_ genotype or the *NFKB2*_rs11574851T_ allele had a significantly increased risk of developing RA whereas a non-significant effect was found in ACPA-negative patients (OR_Rec-ACPA+_ = 1.65, 95%CI 1.04–2.63, *P* = 0.031 vs. OR_Rec-ACPA−_ = 0.86, 95%CI 0.39–1.90, *P* = 0.90 and per-allele OR_ACPA+_ = 1.39, 95%CI 1.06–1.83, *P* = 0.017 and per-allele OR_ACPA−_ = 1.02, 95%CI 0.68–1.52, *P* = 0.93; Table 3). On the other hand, we found that seronegative subjects carrying the *KLRC*_rs7301582T_ or *KLRK1*_rs1049174C_ alleles showed a significantly increased risk of developing RA whereas no effect was detected in ACPA-positive individuals (OR_Dom-ACPA−_ = 1.56, 95%CI 1.18–2.09, P = 0.003 vs. OR_Dom-ACPA+_ = 1.05, 95%CI 0.84–1.30, P = 0.67 and OR_Dom-ACPA−_ = 1.38, 95%CI 1.03–1.84, P = 0.031 vs. OR_Dom-ACPA+_ = 1.09, 95%CI 0.88–1.35, P = 0.42).

**Table 3:** Overall and ACPA-specific associations of NFKB-related polymorphisms and risk of developing RA (discovery population).

Gene	SNP ID	Chr.	Effect allele	Overall RA (n = 2521) 1193 RA/1328 Controls		ACPA^+^ RA patients (n = 1971) 643 RA/1328 Controls		ACPA^-^ RA patients (n = 1593) 265 RA/1328 Controls	
				OR (95% CI)^∂^	*P*	OR (95% CI)^∂^	*P*	OR (95% CI)^∂^	*P*
*GBP6*	rs928655	1	A	0.94 (0.81–1.08)	0.37	0.88 (0.74–1.04)	0.14	1.08 (0.84–1.38)	0.54
*IKBKB*	rs11986055	8	A	0.93 (0.71–1.21)	0.59	1.15 (0.83–1.62)	0.40	0.99 (0.65–1.53)	0.98
*IRF4*	rs1050975	6	A	**1.30 (1.04–1.62)**^**§**^	**0.019**	**1.51 (1.14–1.99)**^**§**^	**0.003**	1.30 (0.91–1.86)^§^	0.15
*IRF4*	rs12203592	6	T	0.97 (0.81–1.18)	0.79	0.99 (0.78–1.24)	0.92	0.83 (0.60–1.17)	0.29
*IRF4*	rs1877175	6	T	1.00 (0.86–1.16)	0.98	0.97 (0.80–1.16)	0.70	1.04 (0.82–1.32)	0.74
*IRF4*	rs7768807	6	T	0.95 (0.83–1.10)	0.51	0.93 (0.78–1.09)	0.36	1.03 (0.82–1.30)	0.78
*KLRC1*	rs7301582	12	T	**1.15 (1.00–1.34)**^**†**^	**0.050**	1.05 (0.84–1.30)^†^	0.67	**1.56 (1.18–2.09)**^**†**^	**0.002**
*KLRK1* | *KLRC4*	rs1049174	12	C	1.18 (0.99–1.41)^†^	0.068	1.09 (0.88–1.35)^†^	0.42	**1.38 (1.03–1.84)**^**†**^	**0.031**
*KLRK1* | *KLRC4*	rs1154831	12	A	1.00 (0.86–1.16)	0.99	1.05 (0.88–1.26)	0.59	0.92 (0.71–1.17)	0.48
*KLRK1* | *KLRC4*	rs2255336	12	A	1.10 (0.94–1.27)	0.22	1.04 (0.87–1.25)	0.68	1.33 (0.99–1.77)^†^	0.055
**LOC105376246**	**rs2722824**	**9**	**A**	**0.96 (0.83–1.10)**	**0.53**	**0.93 (0.79–1.10)**	**0.41**	**1.08 (0.86–1.36)**	**0.50**
*NFKB1*	rs4648110	4	A	1.28 (0.85–1.93)^§^	0.23	**1.65 (1.04–2.63)**^**§**^	**0.031**	0.86 (0.39–1.90)^§^	0.90
*NFKB2*	rs11574851	10	T	1.17 (0.93–1.48)	0.19	**1.39 (1.06–1.83)**	**0.017**	1.02 (0.68–1.52)	0.93
*NFKB2*	rs12769316	10	T	**1.70 (1.04–2.78)**^**§**^	**0.034**	1.70 (0.95–3.06)^§^	0.077	**2.53 (1.24–5.14)**^**§**^	**0.011**
*NFKB2* | *PSD*	rs1056890	10	T	0.96 (0.84–1.09)	0.54	0.95 (0.81–1.12)	0.56	1.01 (0.82–1.25)	0.90
*NFKBIB*	rs3136645	19	C	1.07 (0.91–1.24)	0.42	1.15 (0.95–1.38)	0.14	0.81 (0.62–1.04)	0.10
*NLRP3*	rs4612666	1	T	**1.25 (1.05–1.49)**^**†**^	**0.013**	**1.29 (1.04–1.60)**^**†**^	**0.020**	1.18 (0.89–1.56)^†^	0.26
*REL*	rs13031237	2	T	1.16 (0.91–1.48)^†^	0.24	1.15 (0.85–1.53)^§^	0.36	**1.48 (1.02–2.15)**^**§**^	**0.040**
*REL*	rs842647	2	A	1.08 (0.94–1.24)	0.30	1.10 (0.93–1.31)	0.27	1.05 (0.83–1.33)	0.68
*REL*	rs13017599	2	A	1.06 (0.93–1.20)	0.40	1.04 (0.89–1.21)	0.64	1.17 (0.95–1.43)	0.13
*RELA*	rs11820062	11	T	0.93 (0.82–1.06)	0.29	0.91 (0.78–1.05)	0.20	1.07 (0.88–1.31)	0.49
*RELA*	rs2306365	11	A	1.07 (0.89–1.29)	0.48	1.02 (0.81–1.28)	0.86	1.16 (0.86–1.57)	0.32
*RELA*	rs7119750	11	T	1.09 (0.91–1.32)	0.34	1.04 (0.82–1.30)	0.76	1.24 (0.93–1.65)	0.15
*TLR10*	rs11096957	4	A	1.12 (0.99–1.27)	0.066	1.13 (0.98–1.32)	0.10	1.08 (0.89–1.33)	0.43
*TLR4*	rs4986791	9	T	1.17 (0.89–1.54)	0.25	1.15 (0.83–1.60)	0.40	1.00 (0.63–1.58)	0.99
*TLR5*	rs5744174	1	C	0.99 (0.87–1.13)	0.86	1.03 (0.88–1.20)	0.75	0.89 (0.72–1.10)	0.27
*TLR9* | | *TWF2*	rs187084	3	T	0.97 (0.85–1.10)	0.61	0.93 (0.80–1.09)	0.39	1.02 (0.83–1.25)	0.88
*TRAF1* | | *C5*	rs3761847	9	A	0.97 (0.85–1.10)	0.61	1.00 (0.86–1.17)	0.99	0.91 (0.74–1.13)	0.39

Abbreviations: SNP, single nucleotide polymorphism; OR, odds ratio; CI, confidence interval.

^**∂**^Estimates calculated according to an additive model of inheritance and adjusted for age, sex and country of origin.

^**†**^Estimates calculated according to a dominant model of inheritance and adjusted for age, sex and country of origin.

^§^Estimates calculated according to a recessive model of inheritance and adjusted for age, sex and country of origin.

P ≤ 0.05 in bold. Data on anti-ccp was missing in 285 patients.

Although none of the above-reported associations survived after correction for multiple testing, we attempted to replicate them through meta-analysis of the discovery data with those from the DANBIO registry. The meta-analysis of these two populations, which included 4194 subjects (1888 RA patients and 2306 healthy controls), confirmed that carriers of the *NFKB2*_rs12769316T/T_ genotype had an increased risk of developing RA when compared with those carrying the C allele (OR_Meta_ = 1.78, 95%CI 1.21–2.63, *P* = 0.0037, *I*^2^ = 0.0%, P_Het_ = 0.76; Supplementary Table 2). In addition, although the association was only significant at nominal level (P < 0.05), we also found that carriers of the *NFKB2*_rs11574851T_ allele also had an increased risk of developing RA (OR_Meta_ = 1.29, 95%CI 1.02–1.64, *P* = 0.035, P_Het_ = 0.27). Given that no population stratification was detected (Supplementary Table 3), these findings suggested that the effect attributed to the *NFKB2* locus on the risk of RA was likely true and might depend on a specific haplotype rather than single SNPs. Following this hypothesis, we performed an overall haplotype analysis that revealed that carriers of the *NFKB2*_TC_ haplotype (including the *NFKB2*_rs11574851T_ allele) had a significantly increased risk of developing RA (OR = 2.21, 95%CI 1.37–3.56, P = 0.0011). Although this association did not survive multiple testing correction, it pointed to a role of the *NFKB2*_rs11574851_ SNP to confer risk to RA development.

**Figure 1:**
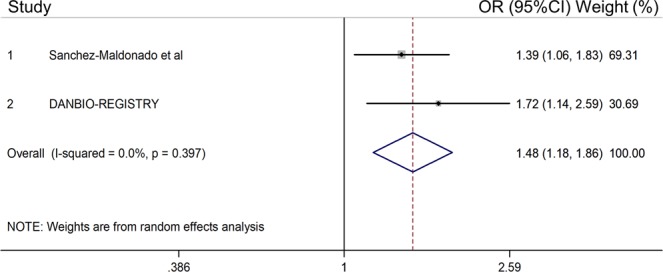
Association of the *NFKB2*_rs11574851_ SNP with the risk of RA in ACPA-positive patients. Association estimates according a random effect model. **P =** **0.0006**.

Most importantly, an ACPA-stratified meta-analysis of our data with those from the DANBIO registry also revealed that each copy of the *NFKB2*_rs11574851T_ allele conferred an additive risk of developing RA in ACPA-positive subjects (OR_Meta_ = 1.48, 95%CI 1.18–1.86, P = 0.0006) that was not detected in ACPA-negative individuals (Table 4 and Fig. 1). Of note, the association of the *NFKB2*_rs11574851_ SNP with an increased risk of RA remained significant after correction for multiple testing and the direction of the effect was consistent with no significant heterogeneity between cohorts (*P*_Het_ = 0.40; Fig. 1). The ACPA-stratified meta-analysis of both populations also showed an increased risk of RA in ACPA-positive and ACPA-negative subjects carrying the *NFKB2*_rs12769316T/T_ genotype (*P* = 0.013 and *P* = 0.004; Table 4 and Supplementary Table 4). Even though none of the associations of the *NFKB2*_rs12769316T/T_ genotype with RA remained significant after correction for multiple testing, these findings supported the notion of a relevant role of the *NFKB2* locus in modulating the RA risk. In order to further confirm this hypothesis, we decided to evaluate whether there was an ACPA-specific haplotype that could influence the risk of developing RA. Interestingly, the ACPA-stratified haplotype analysis including both the discovery and DANBIO cohorts also confirmed that ACPA-positive subjects carrying the *NFKB2*_TT_ haplotype (including the *NFKB2*_rs11574851T_ risk allele) had a significantly increased risk of RA (OR_Haplotype-ACPA+_ = 1.39, 95%CI 1.11–1.74, *P* = 0.0042) whereas no effect was detected in ACPA-negative individuals (OR_Haplotype-ACPA−_ = 1.04, 95%CI 0.75–1.44, *P* = 0.82; Table 5). These results again pointed to an ACPA-specific effect of the *NFKB2* locus to modulate the risk of RA. No additional overall or ACPA-specific associations were confirmed in the meta-analysis of both cohorts.

**Table 4:** Meta-analysis for the association of NFKB- and inflammosome-related polymorphisms and RA risk in ACPA^+^ patients.

Gene	SNP ID	Chr.	Effect allele	Discovery population ACPA^+^ RA vs. controls (n = 1971)		Replication DANBIO Registry ACPA^+^ RA vs. controls (n = 1741)		Meta-analysis ACPA^+^ RA vs. controls (n = 3712)		
				OR (95% CI)^∂^	*P*	OR (95% CI)^∂^	*P*	OR (95% CI)^∂^	*P*	*I*^*2*^
GBP6	rs928655	1	A	0.88 (0.74–1.04)	0.14	1.24 (0.97–1.58)	0.079	1.03 (0.74–1.44)	0.85	0.024
IKBKB	rs11986055	8	A	1.15 (0.83–1.62)	0.40	—	—	—	—	—
IRF4	rs1050975	6	A	**1.51 (1.14–1.99)**^**§**^	**0.003**	0.93 (0.65–1.32)^§^	0.68	1.12 (0.74–1.93)^§^	0.45	0.035
IRF4	rs12203592	6	T	0.99 (0.78–1.24)	0.92	—	—	—	—	—
IRF4	rs1877175	6	T	0.86 (0.75–1.01)	0.065	1.06 (0.84–1.33)	0.61	0.93 (0.77–1.15)	0.52	0.13
IRF4	rs7768807	6	T	0.93 (0.78–1.09)	0.36	—	—	—	—	—
KLRC1	rs7301582	12	T	1.15 (0.97–1.37)	0.096	0.85 (0.67–1.08)	0.19	1.00 (0.74–1.34)	1.00	0.044
KLRK1 | KLRC4	rs1049174	12	C	1.06 (0.90–1.25)	0.45	0.95 (0.76–1.19)	0.66	—	—	—
KLRK1 | KLRC4	rs1154831	12	A	1.05 (0.88–1.26)	0.59	—	—	—	—	—
KLRK1 | KLRC4	rs2255336	12	A	1.04 (0.87–1.25)	0.68	—	—	—	—	—
LOC105376246	rs2722824	9	A	0.93 (0.79–1.10)	0.41	—	—	—	—	—
NFKB1	rs4648110	4	A	1.16 (0.97–1.39)	0.11	—	—	—	—	—
NFKB2	rs11574851	10	T	**1.39 (1.06–1.83)**	**0.017**	**1.72 (1.14–2.59)**	**0.009**	**1.48 (1.18–1.86)**	**0.0006**	0.40
NFKB2	rs12769316	10	T	1.70 (0.95–3.06)^§^	0.077	1.91 (0.93–3.92)^§^	0.080	**1.78 (1.13–2.80)**^**§**^	**0.013**	0.81
NFKB2 | PSD	rs1056890	10	T	0.95 (0.81–1.12)	0.56	—	—	—	—	—
NFKBIB	rs3136645	19	C	1.15 (0.95–1.38)	0.14	—	—	—	—	—
NLRP3	rs4612666	1	T	**1.29 (1.04–1.60)**^**†**^	**0.020**	1.06 (0.81–1.39)^†^	0.68	1.19 (0.99–1.44)^†^	0.072	0.27
REL	rs13031237	2	T	1.15 (0.85–1.53)^§^	0.36	1.15 (0.78–1.70)^§^	0.47	1.15 (0.91–1.45)^§^	0.24	1.00
REL	rs842647	2	A	1.10 (0.93–1.31)	0.27	—	—	—	—	—
REL	rs13017599	2	A	1.04 (0.89–1.21)	0.64	1.02 (0.83–1.25)	0.86	1.03 (0.91–1.17)	0.61	0.88
RELA	rs11820062	11	T	0.91 (0.78–1.05)	0.20	—	—	—	—	—
RELA	rs2306365	11	A	1.02 (0.81–1.28)	0.86	—	—	—	—	—
RELA	rs7119750	11	T	1.04 (0.82–1.30)	0.76	—	—	—	—	—
TLR10	rs11096957	4	A	1.13 (0.98–1.32)	0.10	**0.75 (0.60–0.93)**	**0.010**	0.93 (0.62–1.39)	0.72	**0.002**
TLR4	rs4986791	9	T	1.15 (0.83–1.60)	0.40	—	—	—	—	—
TLR5	rs5744174	1	C	1.03 (0.88–1.20)	0.75	—	—	—	—	—
TLR9 | | TWF2	rs187084	3	T	0.93 (0.80–1.09)	0.39	—	—	—	—	—
TRAF1 | | C5	rs3761847	9	A	1.00 (0.86–1.17)	0.99	—	—	—	—	—

Abbreviations: SNP, single nucleotide polymorphism; OR, odds ratio; CI, confidence interval.

A random effect model was assumed for the meta-analysis of both cohorts.

^**∂**^Estimates calculated according to an additive model of inheritance and adjusted for age and sex.

^**†**^Estimates calculated according to a dominant model of inheritance and adjusted for age and sex.

^§^Estimates calculated according to a recessive model of inheritance and adjusted for age and sex.

P < 0.05 in boldface.

**Table 5:** Overall and ACPA-stratified haplotype association analysis for RA.

NFKB2	rs11574851	rs12769316	99999	Freq	RA patients (n = 4210) OR (95% CI)	*P*	Freq	ACPA-positive patients (n = 3117) OR (95% CI)	*P*	Freq	ACPA-negative patients (n = 2688) OR (95% CI)	*P*
**1**	C	C		0.8181	1.00	—	0.8224	1.00	—	0.8295	1.00	—
**2**	C	T		0.1139	1.14 (0.99–1.31)	0.066	0.1706	1.10 (0.92–1.32)	0.30	0.1088	1.02 (0.79–1.30)	0.91
**3**	T	T		0.0571	1.18 (0.98–1.42)	0.13	0.0530	**1.39 (1.11–1.74)**	**0.0042**	0.0538	1.04 (0.75–1.44)	0.82
**4**	T	C		0.0109	**2.21 (1.37–3.56)**	**0.0011**	—	—	—	—	—	—

^**†**^Estimates calculated according to a dominant model.

Minimum haplotype frequency was set at 0.01. P < 0.05 in bold.

On the basis of the effect found for the *NFKB2*_rs11574851_ or *NFKB2*_rs12769316_ SNPs on the risk of developing RA, we decided to analyse whether these SNPs might exert their biological function directly through the modulation of *NFKB2*-mediated immune responses or indirectly through the regulation of steroid hormone levels. To do that we evaluated if there were any correlation between the *NFKB2*_rs11574851_ and *NFKB2*_rs12769316_ SNPs and levels of 9 cytokines (TNFα, IFNγ, IL1β, IL1RA, IL6, IL8, IL10, IL17, and IL22) after stimulation of whole blood, PBMCs or macrophages with LPS, PHA or Pam3Cys in a cohort of 408 healthy subjects. Although our functional experiments were well powered, we did not find any significant correlation between the *NFKB2*_rs11574851_ and *NFKB2*_rs12769316_ SNPs and cytokine or steroid hormone levels (data not shown). Although these results might suggest no impact of the *NFKB2* variants in modulating immune responses, it is important to mention that we could not evaluate whether the effect of the *NFKB2*_rs11574851_ and *NFKB2*_rs12769316_ SNPs on the modulation of immune responses could be dependent on ACPA status as the genetic analyses indicate.

### Association of selected SNPs with the response to anti-TNF drugs

**Figure 2:**
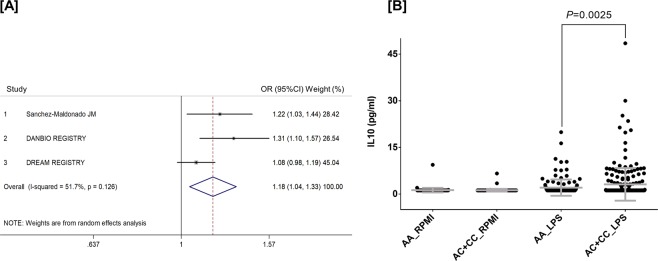
Meta-analysis of the association of the *NFKB2*_rs1056890_ SNP with response to TNFi [**A**] and correlation with higher levels of IL10 after stimulation of PBMCs (n = 377) with LPS [**B**]. [**A**] Association estimates according to a random effect model. ***P***_**Meta**_ = **0.0077**. [**B**] Correlation with IL10 was analysed using genotype data of the *NFKB2*_rs1005044_ SNP, a marker in strong LD with the rs1056890 (r^2^ = 1.00).

When we evaluated the effect of any of the selected SNPs on the response to TNFi (defined as a change in DAS28 after 6 months of treatment), we found a significant effect of the *NFKB2*_rs1056890_ SNP to modulate the response to TNFi at nominal level (P < 0.05). Thus, each copy of the *NFkB2*_rs1056890T_ allele additively increased the drop in DAS28 by 22% after the treatment with TNFi (per-allele OR = 1.22, 95%CI 1.03–1.44, P = 0.025; Table 6). Importantly, when we attempted to replicate this association through a well-powered meta-analysis of our data from the discovery population with those from the DREAM and DANBIO registries (n = 2107), we could confirm that carriers of the *NFKB2*_rs1056890T_ allele showed a significantly higher improvement in DAS28 after treatment with TNFi (OR_Meta_ = 1.18, 95%CI 1.05–1.33, *P* = 0.0077, *I*^2^ = 51.7%, P_Het_ = 0.13; Fig. 2A). Although this association did not remain significant after correction for multiple testing and therefore need to be further validated, this finding suggested that the *NFKB2*_rs1056890_ SNP might modulate the response to anti-TNF drugs through the regulation of the NFKB2-related immune responses.

**Table 6:** Meta-analysis for the association of *NFKB*-related polymorphisms and relative change of DAS28 score (∆DAS28).

Gene	SNP ID	Chr.	Effect allele	Discovery population (n = 604)		Replication DREAM registry (n = 882)		Replication DANBIO Registry (n = 621)		Meta-analysis (n = 2107)		
				OR (95% CI)^∂^	*P*	OR (95% CI)^∂^	*P*	OR (95% CI)^∂^	*P*	OR (95% CI)^∂^	*P*	*I*^*2*^
GBP6	rs928655	1	A	1.05 (0.87–1.27)	0.61	0.90 (0.80–1.00)	0.058	ND	ND	0.95 (0.82–1.10)	0.52	0.17
IKBKB	rs11986055	8	A	0.74 (0.48–1.11)	0.14	0.85 (0.66–1.07)	0.17	0.94 (0.64–1.39)	0.76	0.85 (0.71–1.02)	0.074	0.71
IRF4	rs1050975	6	A	0.95 (0.72–1.24)	0.69	0.99 (0.83–1.17)	0.87	1.24 (0.94–1.65)	0.13	1.03 (0.90–1.18)	0.67	0.33
IRF4	rs12203592	6	T	1.01 (0.77–1.33)	0.93	ND	ND	ND	ND	ND	ND	ND
IRF4	rs1877175	6	T	1.09 (0.90–1.33)	0.37	0.92 (0.82–1.13)*	0.15	0.90 (0.75–1.09)	0.30	0.96 (0.86–1.07)	0.47	0.31
IRF4	rs7768807	6	T	0.86 (0.72–1.03)	0.10	1.04 (0.93–1.16)*	0.52	ND	ND	0.96 (0.80–1.15)	0.65	0.08
KLRC1	rs7301582	12	T	1.05 (0.86–1.27)	0.62	1.00 (0.88–1.12)	0.94	0.99 (0.80–1.22)	0.92	1.00 (0.92–1.11)	0.85	0.90
KLRK1 | KLRC4	rs1049174	12	C	1.08 (0.91–1.29)	0.37	0.96 (0.86–1.08)	0.53	1.07 (0.90–1.27)	0.47	1.01 (0.93–1.10)	0.79	0.42
KLRK1 | KLRC4	rs1154831	12	A	0.89 (0.73–1.10)	0.28	1.05 (0.93–1.19)*	0.40	ND	ND	0.99 (0.84–1.16)	0.88	0.18
KLRK1 | KLRC4	rs2255336	12	A	1.09 (0.90–1.33)	0.38	1.01 (0.89–1.16)	0.81	ND	ND	1.04 (0.93–1.15)	0.54	0.53
LOC105376246	rs2722824	9	A	1.03 (0.86–1.23)	0.77	0.94 (0.85–1.05)	0.32	ND	ND	0.96 (0.88–1.05)	0.41	0.39
NFKB1	rs4648110	4	A	1.07 (0.88–1.29)	0.51	1.00 (0.89–1.13)*	0.95	ND	ND	1.02 (0.92–1.13)	0.71	0.56
NFKB2	rs11574851	10	T	0.97 (0.73–1.29)	0.83	0.92 (0.72–1.18)*	0.53	0.78 (0.57–1.06)	0.11	0.90 (0.76–1.05)	0.18	0.57
NFKB2	rs12769316	10	T	0.92 (0.75–1.13)	0.43	ND	ND	0.86 (0.70–1.06)	0.16	0.89 (0.77–1.03)	0.12	0.65
NFKB2 | PSD	rs1056890	10	T	**1.22 (1.03–1.44)**	**0.025**	1.08 (0.98–1.19)	0.11	**1.31 (1.10–1.57)**	**0.0030**	**1.18 (1.05–1.33)**	**0.0077**	0.12
NFKBIB	rs3136645	19	C	0.90 (0.73–1.11)	0.34	ND	ND	ND	ND	ND	ND	ND
NLRP3	rs4612666	1	T	1.05 (0.87–1.25)	0.62	1.20 (1.05–1.37)*	**0.006**	0.96 (0.80–1.14)	0.62	1.08 (0.94–1.23)	0.28	0.13
REL	rs13031237	2	T	1.07 (0.91–1.26)	0.40	1.03 (0.94–1.14)	0.49	1.08 (0.92–1.28)	0.36	1.05 (0.97–1.13)	0.21	0.86
REL	rs842647	2	A	1.03 (0.86–1.24)	0.72	0.96 (0.87–1.06)	0.45	ND	ND	0.98 (0.89–1.06)	0.57	0.51
REL	rs13017599	2	A	1.07 (0.91–1.27)	0.41	1.03 (0.94–1.14)	0.50	1.03 (0.86–1.21)	0.78	1.04 (0.96–1.12)	0.33	0.92
RELA	rs11820062	11	T	1.07 (0.90–1.26)	0.45	0.92 (0.84–1.01)*	0.081	ND	ND	0.98 (0.84–1.13)	0.74	0.12
RELA	rs2306365	11	A	0.91 (0.71–1.16)	0.45	**1.19 (1.03–1.37)**	**0.021**	ND	ND	1.06 (0.82–1.38)	0.66	0.064
RELA	rs7119750	11	T	0.93 (0.73–1.18)	0.54	ND	ND	ND	ND	ND	ND	ND
TLR10	rs11096957	4	A	1.00 (0.85–1.19)	0.98	0.99 (0.89–1.09)	0.80	ND	ND	0.99 (0.91–1.08)	0.87	0.92
TLR4	rs4986791	9	T	1.15 (0.78–1.70)	0.47	1.18 (0.98–1.41)*	0.077	ND	ND	1.18 (1.00–1.39)	0.056	0.91
TLR5	rs5744174	1	C	0.99 (0.83–1.17)	0.89	ND	ND	ND	ND	ND	ND	ND
TLR9 | | TWF2	rs187084	3	T	1.02 (0.86–1.21)	0.81	0.98 (0.88–1.08)*	0.67	ND	ND	0.99 (0.91–1.08)	0.83	0.69
TRAF1 | | C5	rs3761847	9	A	1.08 (0.91–1.29)	0.37	1.05 (0.95–1.16)	0.33	ND	ND	1.04 (0.96–1.14)	0.35	0.77

Abbreviations: SNP, single nucleotide polymorphism; OR, odds ratio; CI, confidence interval.

A random effect model was assumed for the meta-analysis of both cohorts.

^**∂**^Estimates calculated according to an additive model of inheritance and adjusted for age, sex and country of origin (or age and sex in the replication stages).

*Estimates based on imputed genotypes. P < 0.05 in boldface. No significant heterogeneity (heterogeneity chi-squared) was observed in any meta-analysis reported above.

In order to test this hypothesis, we assessed whether the *NFKB2*_rs1056890_ SNP was associated with cytokine and steroid hormone levels in the HFGP cohort. Although this SNP was not included in the genome-wide association data available from the HFGP cohort, we could evaluate the association of this marker with cytokine and steroid hormone levels through the analysis of neighbouring SNPs in strong LD with it. Our stimulation experiments showed that PBMCs from carriers of the *NFKB2*_rs1005044C_ allele (in complete LD with the rs1056890T allele, r^2^ = 1.00) showed an increased production of IL10 after stimulation of PBMCs with LPS for 24 h (P = 0.0025; Fig. 2B). The analysis of additional neighbouring SNPs belonging to the same LD block allowed us to confirm the association of the rs1056890T allele with increased levels of IL10 (Supplementary Table 5). Although the association of the *NFKB2*_rs1056890_ SNP with a better response to TNFi and its correlation with higher levels of IL10 did not remain statistically significant after correction for multiple testing, altogether these findings point to a role of this marker in determining the response to TNFi likely through the modulation of IL10-mediated immune responses. No significant association of the *NFKB2*_rs1056890_ SNP with response to TNFi was observed when association analysis was stratified by ACPA, which dismissed the implication of ACPA in the functional effect attributed to this polymorphism. We did not find correlation of any of the *NFKB2* SNPs with steroid hormone levels (data not shown), which also ruled out the implication of steroid hormones in the modulation of the IL10-mediated immune responses.

## Discussion

Our data provided, for the first time, evidence that *NFKB2* locus might modulate the risk of RA. The meta-analysis of the data obtained in the discovery population with those from the DANBIO cohort showed a potentially interesting overall association of the *NFKB2*_rs11574851_ SNP with the risk of RA that was further confirmed in an overall haplotype analysis. Most importantly, we found that the effect attributed to the *NFKB2* locus on RA risk depended on the ACPA status. An ACPA-stratified meta-analysis of the discovery and DANBIO populations including 3712 subjects revealed that ACPA-positive subjects carrying the *NFKB2*_rs11574851T_ allele had a significantly increased risk of developing RA whereas no effect was detected in ACPA-negative individuals. Of note, the association of the *NFKB2*_rs11574851T_ allele with an increased risk of RA in ACPA-positive subjects remained significant even after correction for multiple testing and was further confirmed in an ACPA-stratified haplotype analysis that showed that the presence of the *NFKB2*_rs11574851T_ allele was driving the effect of the *NFKB2*_TA_ haplotype on the risk of RA in ACPA positive subjects but not in ACPA-negative individuals.

The *NFKB2* gene is located on chromosome 10q24 and it encodes for a subunit of the *NFKB* complex (p100/p52) that is expressed in multiple immune cells and modulates the inflammation. Other important processes involved in the RA pathology such as Th1 immune responses, activation, abnormal apoptosis and osteoclast differentiation and proliferation^10^ are also impacted. It is broadly known that RA arises as a consequence of the interaction between genetic and environmental factors and that the *NFKB* pathway plays a central role in determining the onset of the disease and its progression. In addition, it has been reported that the genetic and environmental factors that predispose to RA development are substantially different between ACPA-positive and ACPA-negative subjects. Recent studies have demonstrated, for instance, that the effect attributed to the two major genetic risk factors for RA (shared epitope of the HLADRB1 and a SNP on the PTPN22 gene) is clearly dependent on the ACPA status having a more evident effect in ACPA-positive subjects than in those lacking of these antibodies^38^. Furthermore, recent GWAS studies have reported the existence of a completely different genetic component or even a gene-smoking interaction pattern between ACPA-positive and ACPA-negative patients, again suggesting a relevant role of ACPA in determining the onset of the disease^39,40^. However, up to now, little is known about the effect of ACPA on the control of the *NFKB* pathway. Interestingly, recent investigations have demonstrated that the treatment of PBMCs-derived macrophages with ACPA induced the activation of the *NFKB* pathway and subsequently the induction of the NLRP3-inflammasome and the production of pro-inflammatory cytokines^41^. Mechanistically, it was demonstrated that ACPA induces the activation of the *NFKB* pathway through the induction of the interaction between CD147 and integrin β1 or ATGB1, which in turn activates the downstream Akt/NFKB signalling pathway, resulting in the upregulation of NLRP3 and pro-IL-1β expression and further NLRP3 inflammasome activation^41^. Considering these interesting findings, we decided to assess in the HFGP cohort if there was any correlation between the *NFKB2* SNPs and pro- and anti-inflammatory cytokine production after stimulation of whole blood, PBMCs or monocyte-derived macrophages with LPS, PHA or Pam3Cys. We also analysed whether *NFKB2* variants could indirectly affect immune responses through the modulation of steroid hormone levels. Despite the use of a large cohort of healthy subjects from the HFGP cohort, we could not find any significant correlation between the *NFKB2*_rs11574851_ and *NFKB2*_rs12769316_ SNPs and cytokine or steroid hormone levels. Although these results suggested that these variants might not exert their effect on RA risk through the modulation of *NFKB2*- or steroid hormone-mediated immune responses, we could not rule out the possibility of a true effect of these variants on the immune response as their effect might depend on the presence of ACPA (as suggested by our genetic data) or even specific haplotypes. In line with this hypothesis, *in silico* analysis using Haploreg data showed that the *NFKB2*_rs11574851_ and *NFKB2*_rs12769316_ SNPs mapped among histone marks in multiple primary T helper naïve and memory cells and primary B cells from peripheral blood and they were predicted to act as enhancers in T helper memory cells and to change motifs for Po6fu1, AP-4, CEBPB, Mef2 and RP58. Even though these data supported the idea of a role of *NFKB2* variants in modulating immune responses, we think that additional experiments are still needed to determine whether ACPA or specific haplotypes are factors involved in modulating the effect of the *NFKB2* locus on the risk of RA.

Besides the role of the *NFKB2* locus in determining the risk of RA, this study also showed a noticeable impact of the *NFKB2* gene in the modulation of the response to TNFi. In particular, the meta-analysis of the discovery population with data from the DREAM and DANBIO registries, including 2107 RA patients, showed that carriers of the *NFKB2*_rs1056890T_ allele had an improvement in DAS28 after treatment with TNFi. We found that the direction of the effect of the *NFKB2*_rs1056890_ SNP on drug response was consistent across populations and that the effect was statistically significant in 2 of the 3 populations analysed. Although at this point it tempting to speculate that this SNP constitutes a biomarker for good response to TNFi in RA patients that might help to design more individualized treatment strategies, the association did not remain significant after correction for multiple testing and, therefore, need to be confirmed in independent populations. Mechanistically, we found that the presence of neighbouring genetic markers in strong LD with the *NFKB2*_rs1056890_ SNP were associated with increased levels of IL10, suggesting that the *NFKB2* locus might be implicated in modulating IL10-mediated immune responses. Although the association of the *NFKB2*_rs1056890_ SNP with IL10 levels neither survive correction for multiple testing, our results were in agreement with previous studies demonstrating that *NFKB2* unlikely *NFKB1* is implicated in the control of antigen presenting cell function and not in the activation of T and B cells. Likewise, recent studies have also identified genetic polymorphisms within the *NFKB* pathway as genetic biomarkers for response to TNFi in RA^42^ but also other autoimmune diseases^42^, which further supported our hypothesis suggesting a key role of the *NFKB2* gene in modulating the response to TNFi. In addition, *in silico* tools such as Regulome showed that the rs1056890 SNP has a score of 4, which means that this polymorphism could affect transcription factor affinity and DNase peak^43^. Using haploreg it was also suggested that the *NFKB2*_rs1056890_ SNP might play a role in modulating immune responses as it mapped among histone marks in primary T helper naïve and T helper memory cells, T regulatory and primary NK cells and it was predicted to alter binding motifs for NRSF, Sin3Ak-20 and PLAG1. These transcription factors have been implicated in bone-related diseases^44^ and their activation results in up-regulation of multiple target genes including immune-related genes such as macrophage colony stimulator factor (MCSF) and insulin growth factor (IGF)-2.

## Conclusions

In conclusion, this study reports, for the first time, a consistent association of the *NFKB2*_rs11574851_ polymorphism and *NFKB2*_TT_ haplotype with an increased risk of developing RA in ACPA-positive subjects. In addition, this study suggests a possible role of the *NFKB2* locus in the modulation of the response to TNFi. Mechanistically, the functional experiments in the 500FG cohort suggested that the effect attributed to the *NFKB2* gene in the modulation of the response to TNFi might be mediated by IL10-mediated immune responses. However, additional studies are still warranted to shed light into the biological processes that link *NFKB2* SNPs and RA risk and drug response.

## Supplementary information

Supplementary information

## Data Availability

All data used in this project have been meticulously cataloged and archived in the BBMRI-NL data infrastructure (https://hfgp.bbmri.nl/) using the MOLGENIS open source platform for scientific data^45^. This allows flexible data querying and download, including sufficiently rich metadata and interfaces for machine processing (R statistics, REST API) and using FAIR principles to optimize Findability, Accessibility, Interoperability and Reusability^46^. Genetic data from the discovery and DANBIO populations can be accessed at ftp.genyo.es and data from the DREAM registry are available at https://www.synapse.org/#!Synapse:syn3280809/wiki/194735 and https://www.synapse.org/#!Synapse:syn3280809/wiki/194736.
