# Supplementary material for: NFKB2 polymorphisms associate with the risk of developing rheumatoid arthritis and response to TNF inhibitors: Results from the REPAIR consortium
Source: Sci Rep. 2020 Mar 9;10:4316. doi: 10.1038/s41598-020-61331-5 (PMC7062729; doi:10.1038/s41598-020-61331-5)
Supplement: Supplementary file 1 — Supplementary information [file 41598_2020_61331_MOESM1_ESM.pdf]

NFKB2 polymorphisms associate with the risk of developing rheumatoid arthritis and response to TNF inhibitors: Results from the REPAIR consortium

Jose Manuel Sánchez-Maldonado[1,2], Manuel Martínez-Bueno PhD[3], Helena Canhão MD, PhD[4], Rob ter Horst[5], Sonia Muñoz-Peña[1], Ana Moñiz-Díez[1], Ana Rodríguez-Ramos[1], Alejandro Escudero MD, PhD[8], Signe B Sorensen PhD[9,10], Merete L Hetland PhD[11,12], Miguel A. Ferrer MD, PhD[13], Bente Glintborg PhD[11,12], Ileana Filipescu MD, PhD[14], Eva Pérez-Pampin MD, PhD[15], Pablo Conesa-Zamora PhD[16], Antonio García MD, PhD[13], Alfons den Broeder PhD[17], Salvatore De Vita MD, PhD [18], Svend Erik Hove Jacobsen PhD[19], Eduardo Collantes MD, PhD[8], Luca Quartuccio MD, PhD[18], Mihai G. Netea MD PhD[5,6], Yang Li PhD[5,7], João E. Fonseca MD, PhD[20,21], Manuel Jurado MD, PhD[1,2], Miguel Ángel López Nevot MD, PhD[2,22], Marieke J. H. Coenen PhD[17], Vibeke Andersen MD PhD[9,10], Rafael Cáliz MD, PhD[1,2,13], Juan Sainz PhD[1,2]

Supplementary Table 1. Demographic and clinical characteristics of anti-TNF patients.

	**Anti-TNF patients (n=2041)**		
Demographic characteristics	*Discovery Population (n=604)*	*DREAM Registry (n=882)**	*DANBIO Registry (n=555)*
Age (years)	58.75 ± 12.22	54.63 ± 12.80	53.86 ± 13.38
Sex ratio (female/male)	4.08 (485/119)	2.07 (477/230)	2.75 (407/148)
Clinical assessment			
Percentage of patients with RF positivity	420 (69.65)	534 (77.62)	175 (62.50)
*Percentage of ACPA-positive patients**	364 (69.07)	151 (62.14)	309 (71.69)
DAS28 at baseline	5.80 ± 1.25	5.33 ± 1.26	4.74 ± 1.27
Disease duration (years)	16.8 ± 9.78	9.70 ± 9.57	8.53 ± 9.12
Treatments			
First biologic agent			
Infliximab (%)	240 (39.74)	244 (34.46)	142 (25.59)
Etanercept (%)	179 (29.64)	130 (18.36)	175 (31.53)
Adalimumab (%)	163 (26.98)	334 (47.18)	137 (24.68)
Golimumab (%)	22 (03.65)	-	33 (05.95)
Certolizumab (%)	-	-	47 (09.37)
Biosimilar Infliximab (%)	-	-	16 (02.88)

Data are means ± standard deviation or n (%). Abbreviations: RF, rheumatoid factor; ACPA: anti-citrullinated protein antibodies; DAS28, disease activity score; DMARDs, disease-modifying anti-rheumatic drugs.
* Clinical data available in 708 patients (those used to genotype the most interesting markers).
RF and ACPA data were available in 598 and 527 patients in the discovery population.
RF and ACPA data were available in 688 and 243 patients in the DREAM population.
RF and ACPA data were available in 280 and 431 patients in the DANBIO population.

Supplementary Table 2. Meta-analysis for the association of NFKB- and inflammosome-related polymorphisms and RA risk.

				Discovery population (n=2521)		Replication DANBIO Registry (n=1740)		Meta-analysis (n=4261)		
**Gene**	**SNP ID**	**Chr.**	**Effect allele**	**OR (95% CI)[∂]**	***P***	**OR (95% CI)[∂]**	***P***	**OR (95% CI)[∂]**	***P***	***I*²**
GBP6	rs928655	1	A	0.94 (0.81-1.08)	0.37	1.12 (0.92-1.37)	0.25	0.93 (0.82-1.06)	0.29	0.76
IKBKB	rs11986055	8	A	0.93 (0.71-1.21)	0.59	-	-	-	-	-
IRF4	rs1050975	6	A	**1.30 (1.04-1.62)[§]**	**0.019**	0.93 (0.70-1.25)[§]	0.64	1.12 (0.80-1.55)[§]	0.52	0.072
IRF4	rs12203592	6	T	0.97 (0.81-1.18)	0.79	-	-	-	-	-
IRF4	rs1877175	6	T	1.13 (0.76-1.69)[§]	0.54	1.40 (0.86-2.27)[§]	0.17	1.23 (0.91-1.68)[§]	0.19	0.50
IRF4	rs7768807	6	T	0.95 (0.83-1.10)	0.51	-	-	-	-	-
KLRC1	rs7301582	12	T	**1.15 (1.00-1.34)**	**0.050**	0.87 (0.71-1.07)	0.18	1.01 (0.77-1.33)	0.94	**0.030**
KLRK1|KLRC4	rs1049174	12	C	1.11 (0.84-1.46)[§]	0.47	**1.56 (1.03-2.36)[§]**	**0.036**	1.27 (0.92-1.76)[§]	0.15	0.18
KLRK1|KLRC4	rs1154831	12	A	1.00 (0.86-1.16)	0.99	-	-	-	-	-
KLRK1|KLRC4	rs2255336	12	A	1.10 (0.94-1.27)	0.22	-	-	-	-	-
LOC105376246	rs2722824	9	A	0.96 (0.83-1.10)	0.53	-	-	-	-	-
NFKB1	rs4648110	4	A	1.10 (0.95-1.29)	0.20	-	-	-	-	-
NFKB2	rs11574851	10	T	1.18 (0.91-1.52)[†]	0.21	**1.52 (1.05-2.21)[†]**	**0.026**	**1.29 (1.02-1.64)[†]**	**0.035**	0.27
NFKB2	rs12769316	10	T	**1.70 (1.04-2.78)[§]**	**0.034**	**1.93 (1.01-3.66)[§]**	**0.042**	**1.78 (1.21-2.63)[§]**	**0.0037**	0.76
NFKB2|PSD	rs1056890	10	T	0.96 (0.84-1.09)	0.54	-	-	-	-	-
NFKBIB	rs3136645	19	C	1.07 (0.91-1.24)	0.42	-	-	-	-	-
NLRP3	rs4612666	1	T	**1.25 (1.05-1.49)[†]**	**0.013**	1.05 (0.83-1.32)[†]	0.69	1.17 (0.99-1.38)[†]	0.073	0.24
REL	rs13031237	2	T	1.16 (0.90-1.48)[§]	0.24	1.14 (0.82-1.58)[§]	0.44	1.15 (0.95-1.41)[§]	0.16	0.93
REL	rs842647	2	A	1.08 (0.94-1.24)	0.30	-	-	-	-	-
REL	rs13017599	2	A	1.10 (0.86-1.40)[§]	0.44	1.33 (0.96-1.85)[§]	0.090	1.18 (0.97-1.43)[§]	0.10	0.36
RELA	rs11820062	11	T	0.93 (0.82-1.06)	0.29	-	-	-	-	-
RELA	rs2306365	11	A	1.07 (0.89-1.29)	0.48	-	-	-	-	-
RELA	rs7119750	11	T	1.09 (0.91-1.32)	0.34	-	-	-	-	-
TLR10	rs11096957	4	A	1.12 (0.99-1.27)	0.066	**0.80 (0.67-0.96)**	**0.016**	0.95 (0.69-1.33)	0.77	**0.003**
TLR4	rs4986791	9	T	1.17 (0.89-1.54)	0.25	-	-	-	-	-
TLR5	rs5744174	1	C	0.99 (0.87-1.13)	0.86	-	-	-	-	-
TLR9||TWF2	rs187084	3	T	0.97 (0.85-1.10)	0.61	-	-	-	-	-
TRAF1||C5	rs3761847	9	A	0.97 (0.85-1.10)	0.61	-	-	-	-	-

Abbreviations: SNP, single nucleotide polymorphism; OR, odds ratio; CI, confidence interval.
A random effect model was assumed for the meta-analysis of both cohorts.
[∂] Estimates calculated according to an additive model of inheritance and adjusted for age and sex.
[†] Estimates calculated according to a dominant model of inheritance and adjusted for age and sex.
[§] Estimates calculated according to a recessive model of inheritance and adjusted for age and sex.
P<0.05 in boldface.

Supplementary Table 3. Minor allele frequencies (MAF) of the NFKB2 SNPs in healthy controls stratified by country (discovery population) and their comparison with the ones reported in the 1000 genomes.

			Minor allele frequency (MAF) in healthy controls							
**SNP ID**	**Minor allele**	**CEU 1000 Genomes**	**SPAIN**	**P**	**PORTUGAL**	**P**	**ITALY**	**P**	**ROMANIA**	**P**
rs11574851	T	0.056	0.068	0.117	0.057	0.964	0.040	0.623	0.052	0.959
rs12769316	A	0.180	0.178	0.869	0.152	0.211	0.173	0.899	0.179	0.963
rs1056890	A	0.355	0.350	0.756	0.331	0.368	0.380	0.701	0.394	0.260

P values calculated considering the 1000 genomes population as reference.

* Frequencies found in the RA populations used for replication of the associations with anti-TNF response.

Supplementary Table 4. Meta-analysis for the association of NFKB- and inflammosome-related polymorphisms and RA risk in ACPA$^-$ patients.

				Discovery population ACPA$^-$ RA vs. controls (n=1971)		Replication DANBIO Registry ACPA$^-$ RA vs. controls (n=1136)		Meta-analysis ACPA$^-$ RA vs. Controls (n=3107)		
**Gene**	**SNP ID**	**Chr.**	**Effect allele**	**OR (95% CI)[∂]**	***P***	**OR (95% CI)[∂]**	***P***	**OR (95% CI)[∂]**	***P***	***I^2***
GBP6	rs928655	1	A	1.08 (0.84-1.38)	0.54	0.89 (0.65-1.22)	0.47	1.00 (0.83-1.22)	0.98	0.34
IKBKB	rs11986055	8	A	0.99 (0.65-1.53)	0.98	-	-	-	-	-
IRF4	rs1050975	6	A	1.28 (0.92-1.79)	0.14	0.82 (0.54-1.25)	0.36	1.04 (0.68-1.61)	0.85	0.10
IRF4	rs12203592	6	T	0.83 (0.60-1.17)	0.29	-	-	-	-	-
IRF4	rs1877175	6	T	1.04 (0.82-1.32)	0.74	0.96 (0.70-1.32)	0.79	1.01 (0.84-1.22)	0.92	0.69
IRF4	rs7768807	6	T	1.03 (0.82-1.30)	0.78	-	-	-	-	-
KLRC1	rs7301582	12	T	**1.56 (1.18-2.09)**[†]	**0.002**	0.76 (0.50-1.15)[†]	0.20	1.11 (0.55-2.24)[†]	0.78	**0.005**
KLRK1|KLRC4	rs1049174	12	C	1.21 (0.79-1.85)[§]	0.37	**2.57 (1.40-4.71)**[§]	**0.0036**	1.71 (0.82-3.56)[§]	0.35	0.050
KLRK1|KLRC4	rs1154831	12	A	0.92 (0.71-1.17)	0.48	-	-	-	-	-
KLRK1|KLRC4	rs2255336	12	A	1.33 (0.99-1.77)[†]	0.055	-	-	-	-	-
LOC105376246	rs2722824	9	A	1.08 (0.86-1.36)	0.50	-	-	-	-	-
NFKB1	rs4648110	4	A	0.86 (0.39-1.90)[§]	0.90	-	-	-	-	-
NFKB2	rs11574851	10	T	1.02 (0.68-1.52)	0.93	1.63 (0.95-2.80)	0.084	1.24 (0.79-1.96)	0.35	0.18
NFKB2	rs12769316	10	T	**2.53 (1.24-5.14)**[§]	**0.011**	1.91 (0.74-4.92)[§]	0.20	**2.28 (1.30-4.04)**[§]	**0.004**	0.64
NFKB2|PSD	rs1056890	10	T	1.01 (0.82-1.25)	0.90	-	-	-	-	-
NFKBIB	rs3136645	19	C	0.81 (0.62-1.04)	0.10	-	-	-	-	-
NLRP3	rs4612666	1	T	1.15 (0.93-1.44)	0.19	1.05 (0.76-1.44)	0.77	1.12 (0.93-1.34)	0.23	0.65
REL	rs13031237	2	T	**1.48 (1.02-2.15)**[§]	**0.040**	0.82 (0.46-1.45)[§]	0.48	1.15 (0.65-2.04)[§]	0.64	0.091
REL	rs842647	2	A	1.05 (0.83-1.33)	0.68	-	-	-	-	-
REL	rs13017599	2	A	1.17 (0.95-1.43)	0.13	0.90 (0.68-1.19)	0.45	1.05 (0.81-1.35)	0.74	0.14
RELA	rs11820062	11	T	1.07 (0.88-1.31)	0.49	-	-	-	-	-
RELA	rs2306365	11	A	1.16 (0.86-1.57)	0.32	-	-	-	-	-
RELA	rs7119750	11	T	1.24 (0.93-1.65)	0.15	-	-	-	-	-
TLR10	rs11096957	4	A	1.08 (0.89-1.33)	0.43	0.87 (0.64-1.19)	0.38	1.00 (0.82-1.23)	0.98	0.25
TLR4	rs4986791	9	T	1.00 (0.63-1.58)	0.99	-	-	-	-	-
TLR5	rs5744174	1	C	0.89 (0.72-1.10)	0.27	-	-	-	-	-
TLR9||TWF2	rs187084	3	T	1.02 (0.83-1.25)	0.88	-	-	-	-	-
TRAF1||C5	rs3761847	9	A	0.91 (0.74-1.13)	0.39	-	-	-	-	-

Abbreviations: SNP, single nucleotide polymorphism; OR, odds ratio; CI, confidence interval.
A random effect model was assumed for the meta-analysis of both cohorts.
[∂] Estimates calculated according to an additive model of inheritance and adjusted for age and sex.
[†] Estimates calculated according to a dominant model of inheritance and adjusted for age and sex.
[§] Estimates calculated according to a recessive model of inheritance and adjusted for age and sex.
P<0.05 in boldface.

Supplementary Table 5. NFKB2 SNPs in strong LD with the rs1056890 variant and showing correlation with IL10 levels.

Variant	Location	Distance (bp)	r2	D'	Consequence Type	Located in gene(s)	Association with IL10 (PBMCs stimulated with LPS, 1ng/ul)
rs3740418	10:102399176	3837	1.000	1.000	non coding transcript exon variant	NFKB2	P=0.0025
rs72845694	10:102409976	6963	1.000	1.000	intron variant	PSD	P=0.0025
rs1005044	10:102392386	10627	1.000	1.000	regulatory region variant	-	P=0.0025
rs2145308	10:102391242	11771	1.000	1.000	regulatory region variant	-	P=0.0025
rs56238324	10:102390057	12956	1.000	1.000	intergenic variant	-	P=0.0025
rs7077329	10:102402210	803	0.957	1.000	intron variant	NFKB2	P=0.0043
rs6584499	10:102428502	25489	0.935	1.000	intron variant	CUEDC2	P=0.0090

Measurements of steroid hormones

Cortisol, 11-deoxycortisol, androstenedione and 17-hydroxyprogesterone were analyzed by Liquid Chromatography Tandem-Mass Spectrometry (LCMSMS) after protein precipitation and solid-phase extraction as described in Ter Horst et al. (2016)[35] with the following additional compound specific configurations and characteristics. Internal standard [$^{13}C_3$]-cortisol (Isoscience, King of Prussia, PA), $^{2}H_5$-11-deoxycortisol (Isoscience, King of Prussia, PA), [$^{13}C_3$]- androstenedione (Isoscience, King of Prussia, PA) and [$^{13}C_3$]-17-hydroxyprogesterone (Isoscience, King of Prussia, PA) were used. Retention time was 1.46 min, 2.68 min, 3.70 min and 4.78 min for cortisol, 11-deoxycortisol, androstenedione and 17-hydroxyprogesterone respectively. An 9-point calibration curve was used cortisol (Sigma); 11-deoxycortisol (Sigma); androstenedione (Sigma) and 17-hydroxyprogesterone (Sigma). Two transitions (qualitative and quantitative) were monitored. Transitions (Q1>Q3) were m/z 363.4 > 121.1 (25kEV) and m/z 363.4 > 97.1 (34 kEV) for cortisol; m/z 366.4 > 124.1 (25 kEV) and m/z 366.4 > 100.1 (35 kEV) for $^{13}C_3$-cortisol; m/z 347.2 > 97.1 (29 kEV) and m/z 347.2 > 109.1 (31 kEV) for 11-deoxycortisol; m/z 352.3 > 100.1 (31 kEV) and m/z 352.3 > 113.1 (29 kEV) for $^{2}H_5$-11-deoxycortisol; m/z 387.2 > 97.1 (23 kEV) and m/z 387.2 > 109.1 (26 kEV) for androstenedione; m/z 290.2 > 100.1 (21 kEV) and m/z 290.2 > 112.1 (26 kEV) for $^{13}C_3$-androstenedione; m/z 331.3 > 97.1 (31 kEV) and m/z 331.3 > 109.1 (31 kEV) for 17-hydroxyprogesterone; m/z 334.3 > 100.1 (30 kEV) and m/z 334.3 > 112.1 (33 kEV) for $^{13}C_3$-17-hydroxyprogesterone. Dwell time was 100 ms, 40 ms, 100 ms and 60 ms for cortisol, 11-deoxycortisol, androstenedione and 17-hydroxyprogesterone, respectively. The method was linear assessed by CLSI EP6 protocol. Recovery was within 96.5 – 102% cortisol, 98.4 – 104% 11-deoxycortisol, 99.4 – 99.8% androstenedione and 98.6 – 102% 17-hydroxyprogesterone. Total CV for cortisol is 3,6% at 301 nmol/L and 3,1% at 1092 nmol/L. Total CV for 11-deoxycortisol is 5,9% at 2,1 nmol/L and 5,1% at 27 nmol/L. Total CV for androstenedione is 4,7% at 3,2 nmol/L and 4,1% at 23 nmol/L. Total CV for 17-hydroxyprogesterone is 5,6% at 2,6 nmol/L and 5,1% at 95 nmol/L. LOQ was 1.91 nmol/L (13.4% CV), 0.10 nmol/L (10% CV),

0.05 nmol/L (10% CV) and 0.10 nmol/L (10% CV) for cortisol, 11-deoxycortisol, androstenedione and 17-hydroxyprogesterone respectively.

REFERENCES (TABLE 2)

1 Ovejero-Benito, M. C. *et al.* Polymorphisms associated with etanercept response in moderate-to-severe plaque psoriasis. *Pharmacogenomics* **18**, 631-638, doi:10.2217/pgs-2017-0014 (2017).

2 Kichaev, G. *et al.* Leveraging Polygenic Functional Enrichment to Improve GWAS Power. *American journal of human genetics* **104**, 65-75, doi:10.1016/j.ajhg.2018.11.008 (2019).

3 Iwaszko, M. *et al.* Influence of CD94 and NKG2A variants on susceptibility to rheumatoid arthritis and efficacy of anti-TNF treatment. *Joint Bone Spine* **83**, 75-79, doi:10.1016/j.jbspin.2015.06.010 (2016).

4 Iwaszko, M. *et al.* Influence of NKG2D Genetic Variants on Response to Anti-TNF Agents in Patients with Rheumatoid Arthritis. *Genes (Basel)* **9**, doi:10.3390/genes9020064 (2018).

5 Suhre, K. *et al.* Connecting genetic risk to disease end points through the human blood plasma proteome. *Nat Commun* **8**, 14357, doi:10.1038/ncomms14357 (2017).

6 Piotrowski, P., Lianeri, M., Olesinska, M. & Jagodzinski, P. P. Prevalence of the NKG2D Thr72Ala polymorphism in patients with systemic lupus erythematosus. *Mol Biol Rep* **39**, 1343-1347, doi:10.1007/s11033-011-0868-1 (2012).

7 Kabalak, G. *et al.* Association of an NKG2D gene variant with systemic lupus erythematosus in two populations. *Hum Immunol* **71**, 74-78, doi:10.1016/j.humimm.2009.09.352 (2010).

8 Sode, J. *et al.* Confirmation of an IRAK3 polymorphism as a genetic marker predicting response to anti-TNF treatment in rheumatoid arthritis. *Pharmacogenomics J* **18**, 81-86, doi:10.1038/tpj.2016.66 (2018).

9 Bank, S. *et al.* Polymorphisms in the NFkB, TNF-alpha, IL-1beta, and IL-18 pathways are associated with response to anti-TNF therapy in Danish patients with inflammatory bowel disease. *Alimentary pharmacology & therapeutics* **49**, 890-903, doi:10.1111/apt.15187 (2019).

10 Stahl, E. A. *et al.* Genome-wide association study meta-analysis identifies seven new rheumatoid arthritis risk loci. *Nature genetics* **42**, 508-514, doi:10.1038/ng.582 (2010).

11 Eyre, S. *et al.* High-density genetic mapping identifies new susceptibility loci for rheumatoid arthritis. *Nature genetics* **44**, 1336-1340, doi:10.1038/ng.2462 (2012).

12 Gregersen, P. K. *et al.* REL, encoding a member of the NF-kappaB family of transcription factors, is a newly defined risk locus for rheumatoid arthritis. *Nature genetics* **41**, 820-823, doi:10.1038/ng.395 (2009).

13 Ali, F. R. *et al.* An investigation of rheumatoid arthritis loci in patients with early-onset psoriasis validates association of the REL gene. *Br J Dermatol* **168**, 864-866, doi:10.1111/bjd.12106 (2013).

14 Varade, J. *et al.* Analysis of the REL polymorphism rs13031237 in autoimmune diseases. *Annals of the rheumatic diseases* **70**, 711-712, doi:10.1136/ard.2010.134593 (2011).

15 Chen, F. *et al.* Genetic Variation in the REL Gene Increases Risk of Behcet's Disease in a Chinese Han Population but That of PRKCQ Does Not. *PloS one* **11**, e0147350, doi:10.1371/journal.pone.0147350 (2016).

16 Ellinghaus, E. *et al.* Genome-wide meta-analysis of psoriatic arthritis identifies susceptibility locus at REL. *J Invest Dermatol* **132**, 1133-1140, doi:10.1038/jid.2011.415 (2012).
17 Bowes, J. *et al.* Comprehensive assessment of rheumatoid arthritis susceptibility loci in a large psoriatic arthritis cohort. *Annals of the rheumatic diseases* **71**, 1350-1354, doi:10.1136/annrheumdis-2011-200802 (2012).
18 Tang, H. *et al.* TLR10 and NFKBIA contributed to the risk of hip osteoarthritis: systematic evaluation based on Han Chinese population. *Sci Rep* **8**, 10243, doi:10.1038/s41598-018-28597-2 (2018).
19 Vrgoc, G. *et al.* Interleukin-17 and Toll-like Receptor 10 genetic polymorphisms and susceptibility to large joint osteoarthritis. *J Orthop Res* **36**, 1684-1693, doi:10.1002/jor.23823 (2018).
20 Nishikawa, R. *et al.* Genetic prediction of the effectiveness of biologics for psoriasis treatment. *J Dermatol* **43**, 1273-1277, doi:10.1111/1346-8138.13412 (2016).
21 Gebura, K. *et al.* Polymorphisms within Genes Involved in Regulation of the NF-kappaB Pathway in Patients with Rheumatoid Arthritis. *Int J Mol Sci* **18**, doi:10.3390/ijms18071432 (2017).
22 Wang, H., Zhou, S., Zhang, J., Lei, S. & Zhou, J. Correlations between TLR polymorphisms and inflammatory bowel disease: a meta-analysis of 49 case-control studies. *Immunol Res* **67**, 142-150, doi:10.1007/s12026-018-9061-0 (2019).
23 Sode, J. *et al.* Genetic Variations in Pattern Recognition Receptor Loci Are Associated with Anti-TNF Response in Patients with Rheumatoid Arthritis. *PloS one* **10**, e0139781, doi:10.1371/journal.pone.0139781 (2015).
24 Bank, S. *et al.* Polymorphisms in the Toll-Like Receptor and the IL-23/IL-17 Pathways Were Associated with Susceptibility to Inflammatory Bowel Disease in a Danish Cohort. *PloS one* **10**, e0145302, doi:10.1371/journal.pone.0145302 (2015).
25 Bank, S. *et al.* Genetically determined high activity of IL-12 and IL-18 in ulcerative colitis and TLR5 in Crohns disease were associated with non-response to anti-TNF therapy. *Pharmacogenomics J* **18**, 87-97, doi:10.1038/tpj.2016.84 (2018).
26 Loft, N. D. *et al.* Associations between functional polymorphisms and response to biological treatment in Danish patients with psoriasis. *Pharmacogenomics J* **18**, 494-500, doi:10.1038/tpj.2017.31 (2018).
27 Oliveira-Tore, C. F. *et al.* Genetic Polymorphisms of Toll-like receptors 2 and 9 as Susceptibility Factors for the Development of Ankylosing Spondylitis and Psoriatic Arthritis. *J Immunol Res* **2019**, 1492092, doi:10.1155/2019/1492092 (2019).
28 Yi, X., Xu, E., Xiao, Y. & Cai, X. Evaluation of the Relationship Between Common Variants in the TLR-9 Gene and Hip Osteoarthritis Susceptibility. *Genet Test Mol Biomarkers* **23**, 373-379, doi:10.1089/gtmb.2019.0010 (2019).
29 Zheng, M. *et al.* Association between TLR-9 gene rs187084 polymorphism and knee osteoarthritis in a Chinese population. *Bioscience reports* **37**, doi:10.1042/BSR20170844 (2017).
30 Huang, C. M. *et al.* Association of toll-like receptor 9 gene polymorphism in Chinese patients with systemic lupus erythematosus in Taiwan. *Rheumatol Int* **32**, 2105-2109, doi:10.1007/s00296-011-1925-8 (2012).
31 Bank, S. *et al.* Polymorphisms in the inflammatory pathway genes TLR2, TLR4, TLR9, LY96, NFKBIA, NFKB1, TNFA, TNFRSF1A, IL6R, IL10, IL23R, PTPN22, and PPARG are associated with susceptibility of inflammatory bowel disease in a Danish cohort. *PloS one* **9**, e98815, doi:10.1371/journal.pone.0098815 (2014).

32 Cho, W. K. *et al.* Association of Polymorphisms in Toll-Like Receptors 4 and 9 with Autoimmune Thyroid Disease in Korean Pediatric Patients. *Int J Endocrinol* **2017**, 2304218, doi:10.1155/2017/2304218 (2017).

33 Bank, S. *et al.* Associations between functional polymorphisms in the NFkappaB signaling pathway and response to anti-TNF treatment in Danish patients with inflammatory bowel disease. *Pharmacogenomics J* **14**, 526-534, doi:10.1038/tpj.2014.19 (2014).

34 Plenge, R. M. *et al.* TRAF1-C5 as a risk locus for rheumatoid arthritis--a genomewide study. *The New England journal of medicine* **357**, 1199-1209, doi:10.1056/NEJMoa073491 (2007).
